# PLG-ViT: Vision Transformer with Parallel Local and Global Self-Attention

**DOI:** 10.3390/s23073447

**Published:** 2023-03-25

**Authors:** Nikolas Ebert, Didier Stricker, Oliver Wasenmüller

**Affiliations:** 1 Research and Transfer Center CeMOS, Mannheim University of Applied Sciences, 68163 Mannheim, Germany; o.wasenmueller@hs-mannheim.de; 2 Department of Computer Science, RPTU Kaiserslautern-Landau, 67663 Kaiserslautern, Germany; didier.stricker@dfki.de

**Keywords:** transformer, self-attention, image classification, object detection, semantic segmentation

## Abstract

Recently, transformer architectures have shown superior performance compared to their CNN counterparts in many computer vision tasks. The self-attention mechanism enables transformer networks to connect visual dependencies over short as well as long distances, thus generating a large, sometimes even a global receptive field. In this paper, we propose our Parallel Local-Global Vision Transformer (PLG-ViT), a general backbone model that fuses local window self-attention with global self-attention. By merging these local and global features, short- and long-range spatial interactions can be effectively and efficiently represented without the need for costly computational operations such as shifted windows. In a comprehensive evaluation, we demonstrate that our PLG-ViT outperforms CNN-based as well as state-of-the-art transformer-based architectures in image classification and in complex downstream tasks such as object detection, instance segmentation, and semantic segmentation. In particular, our PLG-ViT models outperformed similarly sized networks like ConvNeXt and Swin Transformer, achieving Top-1 accuracy values of 83.4%, 84.0%, and 84.5% on ImageNet-1K with 27M, 52M, and 91M parameters, respectively.

## 1. Introduction

In the last decade, deep convolutional neural networks (CNNs) [1,2,3] have emerged as one of the standards in computer vision. A critical point in the development of new architectures has always been the receptive field, i.e., the area of the input on which the output values depend. Various methods, such as dilated [4,5] or deformable convolutions [6], attempt to enlarge the receptive field while maintaining complexity and weights. However, in most cases the field remains limited to (semi-)local areas. Recently, Dosovitskiy et al. [7] introduced the first Vision Transformer (ViT), adapting the concept of self-attention [8] to achieve a global receptive field processing non-overlapping image patches. This attention mechanism allows the modeling of dependencies over long spatial distances and has led to transformers surpassing CNNs [1,2,9] in various vision tasks [7,10,11], especially image classification.

Inspired by ViT, several transformer architectures [12,13,14,15,16] have been introduced to further improve the accuracy and efficiency for various tasks. In general, these architectures can be divided into local and global approaches. On the one hand, global approaches (e.g., Pyramid Vision Transformer (PVT) [13]) usually retain the global receptive field of ViT, but decrease the resolution of the key and value feature maps to reduce complexity. However, the complexity of these models is often still quadratic to the resolution of the input image, leading to challenges for high-resolution images. On the other hand, local approaches (e.g., Swin Transformer [12]) use non-overlapping windows, slowly increasing the receptive field by window shifting to describe interactions between different stages. As a result, the ability of the self-attention to capture long-range information is limited. Recently, approaches to combine global as well as local receptive fields have been presented [15,16,17,18], usually with added architectural complexity and computational costs.

**Figure 1:**
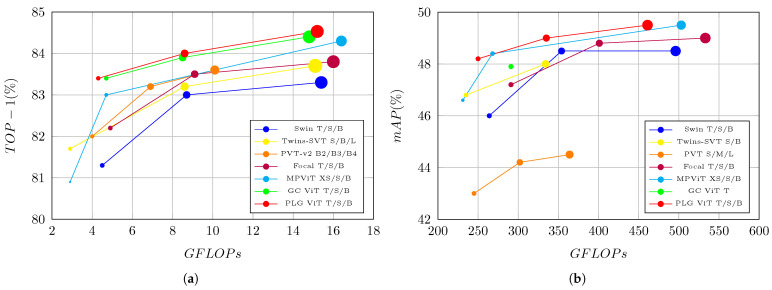
Comparison of PLG-ViT in terms of accuracy and efficiency with state-of-the-art methods [12,13,15,16,17,19,20] on the benchmarks ImageNet [21] and COCO [22]. Each method is represented by a circle whose diameter is proportional to the number of parameters. Our PLG-ViT outperforms comparable methods in terms of accuracy with similar numbers of FLOPs and parameters on both benchmarks. (**a**) Classification on ImageNet [21]. (**b**) Object Detection on COCO [22].

Thus, we propose our novel hierarchical Parallel Local-Global Vision Transformer (PLG-ViT), a general-purpose backbone that has a local as well as global receptive field beginning from its first stage, but without adding architectural complexity or computational costs. We achieve this with our efficient local-global self-attention mechanism with multiple receptive fields at each stage for the parallel processing of fine-grained local as well as coarse-grained global features. Furthermore, we present a light-weighted patch-sampling technique to generate representative global tokens of a fixed window-size and a novel convolution-based feed-forward network (CCF-FFN) for an extra inductive bias during the forward-path for each self-attention operation. Finally, a comprehensive evaluation is presented on various computer vision benchmarks to demonstrate the superior performance of our PLG-ViT (see Figure 1).

## 2. Related Works

Transformers were first introduced in natural language processing for machine translation [8] and quickly displaced LSTMs as the state-of-the-art method in this area. The main reason for this is the multihead self-attention mechanism, which flexibly models the relationship of individual input tokens, even over long distances.

In the field of computer vision, transformers were first presented by Vision Transformer (ViT) [7], directly applying transformer-encoders [8] on non-overlapping image patches for classification. Further, ViT achieves an impressive trade-off in speed and accuracy for the task of image classification when compared to classic convolutional networks (CNNs) [1,2,3]. In contrast to CNNs, ViT has a global receptive field, which can be used to capture long-range spatial image dependencies and is also free of an inductive bias. However, the major drawbacks of ViT are the need for large-scale datasets (e.g., JFT-300M [23]), the computational quadratic complexity, and the slow convergence during training. To overcome most of these drawbacks, DeIT [14] introduced different strategies for data-efficient training, making it possible to effectively train ViT on smaller datasets such as ImageNet-1K [21]. Further extensions [24,25,26,27,28] of ViT were also presented to improve the classification accuracy.

The mentioned methods work well for image classification, but are less suitable as general-purpose backbones for dense downstream tasks. This can be attributed to the lack of downsampling and the generation of single-resolution feature maps. In dense tasks such as object detection, multi-scale feature pyramids [29] are utilized to accomplish scale-invariant recognition of objects and to achieve state-of-the-art results. In addition, the high computational cost of the self-attention technique for high-resolution images is a significant challenge due to the quadratic increase in complexity with image size. To overcome these issues and make transformers suitable for downstream tasks, several methods [12,13,15,16,19,30] adapt the pyramid structures of CNNs [1,3]. Pyramid Vision Transformer (PVT) [13] and Swin Transformer [12] were the first approaches in which transformer-based hierarchical architectures were utilized for dense computer vision tasks. PVT retains the global receptive field of the original ViT, but decreases the resolution of the key and value matrices to reduce the model complexity. PVTv2 [19] further improves accuracy and efficiency compared to the original PVT by adding a convolutional feed-forward network, linear attention, and overlapping patch embedding. For both, the complexity is still quadratic to the resolution of the image. In comparison, Swin Transformer introduces non-overlapping window partitions and performs self-attention for each local window. This results in a linear complexity to the number of input tokens. For communication between each window, Swin performs window shifting for the subsequent transformer-layers. Initially, these designs support only local receptive fields within the attention. Similar to a CNN, the resulting effective receptive field enlarges with every transformer layer, eventually encompassing the entire image, but limiting the ability of self-attention to grasp long-range dependencies. Furthermore, the window shifting is not optimized for use on GPUs and proves to be memory-inefficient [16].

In addition to methods that use global [7,13,14] or local [12,31,32] receptive fields, there are also first methods [15,16,17,18] that target a combination of both. For example, Focal Transformer [15] introduces focal self-attention to incorporate fine-grained local and coarse-grained global interactions. However, this is only achieved with a very complex architecture in conjunction with a high computing effort. Another approach is DAT [18], which uses a complex network-in-network structure to determine the key and value pair depending on the data in the way of deformable convolutional networks [6]. Multi-Path ViT [20] embeds features of the same size with patches of different scales by using overlapping patch embedding. Then, tokens of different scales are fed into the transformer encoders via multiple paths. The resulting features are concatenated and connect fine and coarse feature representations at the same feature level. Global Context ViT [17] generates the global receptive field via alternating global and local query tokens. This means that each layer can capture either exclusively local or exclusively global features.

In our approach, we implement the parallel generation of local and global features within each layer. These features are combined in a learned manner by the feed-forward part of the PLG-ViT block, removing the need for complex fusion of these features. Our method allows the extraction of local information in a global context through the network, while efficiently generating global as well as local receptive fields. This keeps the complexity of the model manageable when using high-resolution images for sophisticated downstream tasks such as object detection.

## 3. PLG-ViT Architecture

**Figure 2:**
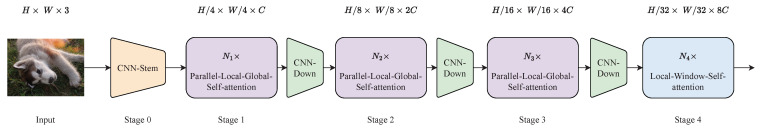
Architecture of our PLG-ViT. Overlapping patches are generated by a CNN stem followed by 3 stages of our parallel local-global self-attention blocks. In the last stage, only local features are extracted. Between the individual stages, convolutional downsampling generates overlapping patches.

The hierarchical framework of the proposed PLG-ViT for obtaining multi-scale features is presented in Figure 2. The structure of the proposed method follows the model of established convolutional networks (CNNs) [1,2,3] and transformers [12,13,16,33]. We reduce the spatial resolution of the input and in return increase the depth of the features during propagation through the network. Furthermore, our work focuses on the parallel extraction of global and local features, which are subsequently fused together by our convolutional feed-forward network. Due to the different receptive fields, a wide variety of semantic and representative features are extracted for further processing.

**Figure 3:**
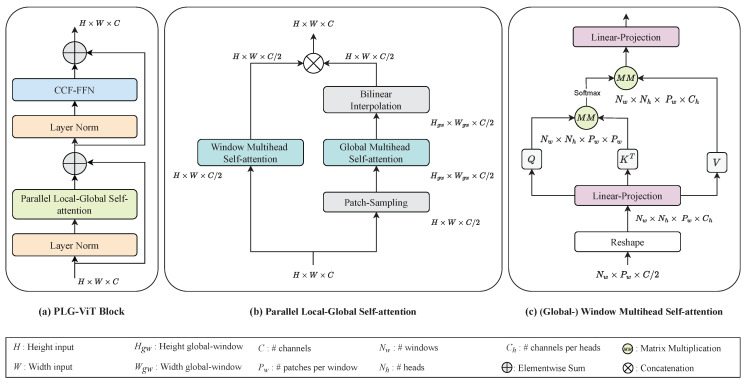
Model architecture of our PLG-SA block. The PLG-ViT block consists of the parallel local-global self-attention and the convolutional feed-forward network (CCF-FFN). The parallelism of window self-attention and global self-attention enables efficient as well as effective recognition of fine-grained local in addition to coarse-grained global features and interactions in the image.

To obtain features with different spatial resolutions, we divide the model into five stages, with the last four stages consisting of transformer layers. At the first stage, overlapping patches of a given input-image with the resolution $z\in R^{H\times W\times 3}$ are generated from a CNN stem inspired by GC ViT [17]. This CNN stem with a total stride of s=4 projects the patches into a *C*-dimensional embedding space, generating the input of the first transformer-stage with a shape of $z\in R^{\frac{H}{4}\times \frac{W}{4}\times C}$. This transformer stage consists of $N_{1}\times$ proposed PLG blocks as shown in Figure 3a, which extract and merge local as well as global features in parallel. After each transformer stage, the spatial resolution of the output features is reduced and the channel size is increased by a factor of 2. Transformer stages 2 and 3 have an identical layout to stage 1. The final stage 4 performs only local self-attention due to the low spatial resolution of the features at this stage.

### 3.1. Parallel Local-Global Self-Attention

As mentioned earlier, PLG blocks for parallel local-global self-attention (PLG-SA) are the core element of our model and are presented in Figure 3a. The structure was inspired by the original transformer-encoder block [7,8], but we replaced standard multihead self-attention with a parallel local-global self-attention operation. This allows the global analysis of the image complemented by a local view. Furthermore, our CCF feed-forward network (CCF-FFN) replaces the linear MLP of the original transformer for further improvements in accuracy (see Section 4.4). Before self-attention and CCF-FFN, layer normalization [34] is performed. The *i*-th PLG block can be described by

(1) $$\begin{array}{cc} z_{i}^{*} & =\mathrm{PLG}-\mathrm{SA}\left(\mathrm{LN}(z_{i-1}\right))+z_{i-1},\\ z_{i} & =\mathrm{CCF}-\mathrm{FFN}\left(\mathrm{LN}(z_{i}^{*}\right))+z_{i}^{*}, \end{array}$$

where LN refers to layer normalization.

For efficient processing of local and global features, we perform parallel local and global self-attention (PLG-SA) as shown in Figure 3b. For this purpose, we assume that the input features of the PLG-SA have the shape $z\in R^{H\times W\times C}$, where *H* and *W* indicate the spatial dimension of the features and *C* refers to the feature depth. In the first step of PLG-SA, we split the input *z* along the feature depth and generate the local features $z_{l}\in R^{H\times W\times \frac{C}{2}}$ and the global features $z_{g}\in R^{H\times W\times \frac{C}{2}}$. By splitting the feature maps, the number of calculations is decreased, which reduces the model complexity in terms of FLOPs and parameters. In contrast to well-known approaches such as PVT [13], our self-attention mechanism has a linear complexity to the image resolution instead of a quadratic one. More details about the complexity in terms of image size can be found in Section 4.5.

**Figure 4:**
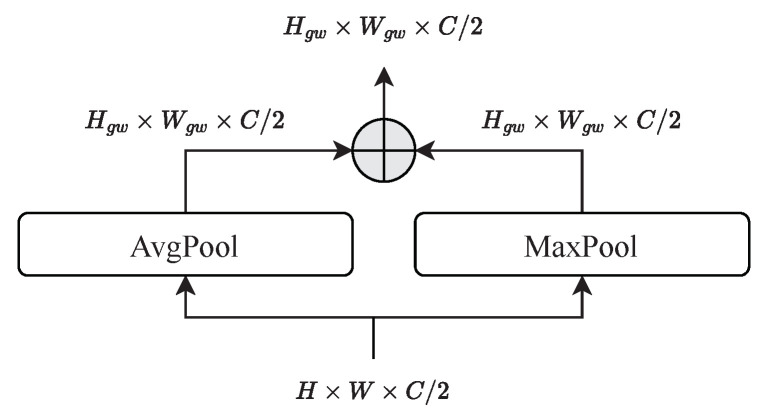
Patch-sampling to generate global windows. Patch-sampling consists of max- and average-pooling, which are added together at the end.

To create windows with a spatially limited receptive field for fine-grained features, we follow the window partitioning strategy of Swin Transformer [12]. This allows us to apply multihead self-attention to the local feature maps $z_{l}$ (see Figure 3c). For global self-attention, we first perform the patch-sampling operation illustrated in Figure 4. Patch-sampling performs adaptive max- and average-pooling to the global features $z_{g}$ and reduces the spatial resolution to $z_{g}^{*}\in R^{H_{gw}\times W_{gw}\times \frac{C}{2}}$, where $\left(H_{gw},W_{gw}\right)$ refers to the global window-size. Due to the combination of average- and max-pooling, which is inspired by attention blocks such as CBAM [35], we are able to extract a rich feature description of each image region. In effect, a single window with a global receptive field is created, to which multihead self-attention is subsequently applied. The self-attention for local and global self-attention are computed as

(2) $$\mathrm{Attention}\left(q,k,v\right)=\mathrm{Softmax}\left(\frac{qk^{T}}{\sqrt{d}}+b\right)v,$$

where q,k,v are query, key, and value matrices; *d* is a scaling factor; and *b* is a trainable relative position bias term [36,37]. As shown in Section 4.4, a relative position bias *b* improves the accuracy, especially for downstream tasks such as object detection. After applying self-attention to $z_{g}^{*}$, a bilinear interpolation is performed to recover the original spatial resolution of $z_{g}$. Finally, the local $z_{l}$ and global $z_{g}$ features are concatenated again to $z^{*}\in R^{H\times W\times C}$. Due to the fusion of local and global features, we are able to generate representative and highly semantic feature maps for later usage in different sparse and dense downstream tasks.

### 3.2. Additional Blocks

Inspired by MixFFN [11], we implement a convolution-based feed-forward network (FFN) that combines fully connected and convolutional layers. As shown in Section 4.4, an FFN with an inductive bias of convolutional layers enables the transformer to encode position and local information, further improving accuracy. Our CCF-FFN consists of a 1×1 point-wise convolution (PWConv) to expand the dimensions of the input $z_{in}$ by the ratio of α=4 followed by a 3×3 depth-wise convolution (DWConv). Finally, summation is performed with the inputs immediately after applying a last fully-connected layer (FC) to the features. The complete CCF-FFN is formulated as

(3) $$\begin{array}{cc} z^{*} & =\mathrm{GeLU}(\mathrm{LN}\left(\mathrm{PWConv}\left(z_{in}\right)\right)),\\ z^{*} & =\mathrm{GeLU}(\mathrm{LN}\left({\mathrm{DWConv}}_{3\times 3}\left(z^{*}\right)\right)),\\ z_{out} & =\mathrm{FC}\left(z^{*}\right)+z_{in}, \end{array}$$

where LN refers to layer normalization and GeLU refers to Gaussian error linear units [38]. For downsampling we use a modified version of Fused-MBConv [17,39]. The complete downsampling can be described by

(4) $$\begin{array}{cc} z^{*} & =\mathrm{SE}(\mathrm{GeLU}\left({\mathrm{DWConv}}_{3\times 3}\left(z_{in}\right)\right)),\\ z_{out} & =\mathrm{LN}({\mathrm{SConv}}_{3\times 3}\left(\mathrm{PWConv}\left(z^{*}\right)+z_{in}\right)), \end{array}$$

where LN, GeLU, and SE denote layer normalization, Gaussian error linear units, and a squeeze and excitation block [40]. ${\mathrm{SConv}}_{3\times 3}$ refers to a 3×3 convolutional layer with a stride of 2. For CNN-Stem, we add an additional strided 3×3 convolutional layer in front of the complete downsampling operation.

### 3.3. Architecture Variants

In this paper, we consider three network configurations: PLG-ViT *Tiny*, *Small,* and *Base*; these are similar to related methods [1,12,13,33]. The *Tiny* and *Small* versions are only 0.25× and 0.5× the size and computational complexity of PLG-ViT *Base*, respectively. We set the local and global window sizes to 7 and 14 by default. A more detailed analysis of the impact of the global window size can be found in Section 4.4. The dimension *d* of each head is 32 for *Base* and *Tiny*, and 24 channels for PLG-ViT *Small*. The expansion ratio of CCF-FFN is α=4 for all experiments. The other hyperparameters of the three model variants are

- *Tiny*: *C* = 64, layer numbers = {3, 4, 16, 4}, *d* = 32
- *Small*: *C* = 96, layer numbers = {3, 3, 16, 3}, *d* = 24
- *Base*: *C* = 128, layer numbers = {3, 3, 16, 3}, *d* = 32,

where *C* is the channel number of the hidden layers in the first transformer-stage, which doubles for each subsequent stage.

## 4. Evaluation

In the following evaluation, we demonstrate the usability of our network in general computer vision tasks. Therefore, we perform comprehensive experiments on the benchmarks ImageNet-1K [21] for image classification, COCO [22] for object detection and instance segmentation, and ADE20K [41] for semantic segmentation. Domains such as autonomous driving [42,43] and medical technology [44,45] are some of the most important areas for the application of computer vision tasks. For this reason we also investigate the effectiveness of our network in these domains using the two datasets BDD100K [46] and AGAR [45]. In the following, a comparison of our method with the state-of-the-art is conducted. Then, the individual network components are examined in the context of an ablation study. Visual examples of the individual tasks and more detailed explanations of the different training strategies are presented in Appendix C.

### 4.1. Image Classification

For the task of image classification we use ImageNet-1K [21], which consists of 1.28M images for training and 50K images for validation, including 1000 classes. The classification task is solved by combining global average pooling of the output features of the last transformer-stage with a subsequent linear classifier. For evaluation of the Top-1 accuracy, we report the results on a single crop and use an identical configuration to Swin Transformer [12]. To allow a fair comparison, we have only listed methods of similar size and complexity. There are various approaches [31,47,48] that achieve significantly higher accuracy using more parameters, more FLOPs, additional data, and pre-training strategies. However, these methods are not considered in the following evaluation.

We report our results on ImageNet-1K validation in Table 1 after training for 300 epochs. As can be seen, our PLG-ViT achieves significant improvements on Top-1 accuracy with a similar number of parameters and model complexity (FLOPs). We are able to outperform established state-of-the-art methods like Pyramid Vision Transformer (PVT) v1/v2 [13,19], Swin Transformer [12], and Focal Transformer [15] at all three scales. Specifically, PLG-ViT outperforms its Swin counterparts by +2.1, +0.7, and +1.0 Top-1 accuracy for *Tiny*, *Small*, and *Base* model configurations, respectively. GC ViT [17] and our model are on par in terms of Top-1 accuracy. However, we will show later that we are able to outperform GC ViT in the tasks of object detection (see Section 4.2) and semantic segmentation (see Section 4.3). Our PLG-ViT also surpasses SoTA-CNNs (e.g., ConvNeXt [9]). For the *Tiny* version of our network, we have also listed the results for a smaller window size of 7 instead of 14. Even in this case, our network shows competitive results. A comparison of image classification Top-1 accuracy in terms of model complexity (i.e., FLOPs) and number of parameters is visualized in Figure 1a.

### 4.2. Object Detection and Instance Segmentation

For training and evaluation of object detection and instance segmentation, we utilize mainly the COCO [22] dataset, which contains 118K training and 5K validation images of everyday objects from 80 classes. Our pre-trained models are used as backbones for the typical frameworks of Faster RCNN [55] and RetinaNet [33] for pure object detection, and Mask RCNN [56] for instance segmentation. Like most competing methods [12,16,17,33], we follow the two standard schedules, a 1× schedule with 12 epochs and single-scale inputs and a 3× schedule with 36 epochs and multi-scale inputs. The implementation of all methods is based on the MMDetection Framework [57].

In Table 2 we report the results in terms of mAP for bounding boxes (AP^box^) and segmentation masks (AP^mask^) of our PLG-ViT Mask RCNN model with a 3× training schedule and multi-scale training. It can be seen that our transformer is able to outperform all CNN-based methods by 2.0 up to 6.2 AP^box^ and 1.2 to 5.0 AP^mask^. The network also performs very well in comparison with the other vision transformers. PLG-ViT outperforms its Swin Transformer [12] counterparts from 0.5/0.2 to 2.0/1.3 AP^box^/AP^mask^ at comparable settings. Even though GC ViT-T [17] and our PLG ViT-T are on par in terms of image classification accuracy, our model is able to outperform it by 0.3 AP^box^ for object detection. In terms of the accuracy of instance segmentation (AP^mask^), our method is outperformed by MPViT. However, a comparison of AP^box^ in terms of model complexity (i.e., FLOPs) and number of parameters is visualized in Figure 1b. Our method demonstrates the best tradeoff in terms of complexity to accuracy, even compared to MPViT.

To prove the universal applicability of our network, we investigated the performance for object detection in different domains with diverse characteristics. For this purpose, training of our PLG-ViT *Tiny* as the backbone of RetinaNet [33] and Faster RCNN [55] on the three datasets COCO [22], BDD100K [46], and AGAR [45] took place. BDD10K shows daily road scenarios and contains 70K images for training and 10K images for validation. In comparison, the AGAR dataset from the field of medical technology shows high-resolution images of five different bacterial colonies that grew on a culture medium of agar plates and contains approximately 5K training as well as 2K validation images. Single-scale training was performed for 12 epochs with the described settings of a 1× scheduler. We compared the performance of our model with the CNN-based ResNet50 [1] and ConvNeXt-T [9], as well as the transformer backbones Swin-T [12] and PVTv2-b2 [19].

The results of this domain analysis can be found in Table 3. We report the AP^box^ for different IoU thresholds and object sizes. It can be clearly seen that our network performs better in the relevant metric of the AP than the comparative methods with a comparable number of parameters and model complexity, regardless of the detector used.

These experiments demonstrate the efficiency of our network in terms of high resolution input images. Especially in the field of medical data, as the example of the AGAR dataset shows, low complexity and fewer parameters are a big advantage, because the images have a particularly high resolution in order to represent even the smallest objects. To ensure the applicability of the network even without expensive high-end GPUs, it is important to consider the complexity in terms of FLOPs and the number of parameters in the design of the network. In Section 4.5, we discuss the relationship between resolution and network complexity in more detail.

### 4.3. Semantic Segmentation

For benchmarking our results for semantic segmentation we used the ADE20K [41] dataset, which contains 20K images for training and 2K images for validation from 150 classes. We employed our pre-trained model as the backbone and utilized UPerNet [58] in MMSegmentation [59] as the framework of choice. For fair comparison, all settings were identical to Swin [12].

The training results after 160K iterations are reported in Table 4 for single- and multi-scale evaluation. PLG-ViT is able to outperform Swin Transformer [12] counterparts by 1.9, 0.4, and 1.8 mIoU for *Tiny*, *Small*, and *Base* models, respectively. For the *Tiny* and *Small* model sizes, we slightly lag behind competing approaches [9,17,18,20] in terms of mIoU. However, our *Base* model is able to outperform most competing methods of the same complexity by a margin up to 1.8 and 1.0 mIoU for single- and multi-scale testing, respectively. Only MPViT [20] achieves slightly better results (+0.4 mIoU) for single-scale testing.

### 4.4. Ablation Study

In this section, we ablate the most important design elements and modules of the proposed PLG-ViT. We use ImageNet-1K [21] for image classification and COCO [22] instance segmentation utilizing Mask RCNN [56] with a 1× scheduler and multi-scale training. Ablation on the effectiveness of several components is reported in Table 5.

**PLG-SA.** First, we investigated the network performance without our novel parallel local-global self-attention, which is described in Section 3.1. A slight decrease in accuracy on ImageNet (−0.2 Top-1) and COCO (−0.2 AP^box^ and −0.1 AP^mask^) can be seen, with an increase in computational complexity of about 15%. Due to the use of convolutional layers within the model, a communication of all pixels can be performed even with static non-overlapping windows during self-attention.

**CCF-FFN.** Then, the effect of our novel CCF-FFN (see Section 3.2) was investigated. We observed that the inductive bias of the convolutional operations that are applied in the feed-forward network (FFN) is crucial for the performance on ImageNet and COCO. With the CCF-FFN we gained +0.8 Top-1 accuracy on ImageNet and +2.5 AP^box^/+1.2 AP^mask^ on the COCO benchmark.

**Convolutional patch-embedding (Conv-PE).** We further investigated the impact of the convolutional and overlapping patch-embedding and downsampling (see Section 3.2). As reported in Table 5, there is a slight decrease of −0.1 in ImageNet Top-1 accuracy. However, a −0.5 decrease in AP^mask^ and AP^box^ is noticeable. This indicates that convolutional downsampling is important for complex downstream tasks such as detection and segmentation.

**Network Configuration.** First, we picked the network parameters following the example of Swin Transformer [12]. For example, we chose a layer depth of [2,2,6,2] and an initial channel size of 96 for PLG-ViT *Tiny*. Through a series of experiments, we adjusted the layer depth to [3,4,16,4] and the initial channel size to 64m, as described in Section 3.3. The training results for ImageNet and COCO following the original Swin Transformer network configuration are listed in Table 5. A slight decrease in Top-1 accuracy of −0.4 and a broad decrease of −1.6 AP^box^ on COCO are reported.

**Relative position bias.** Next, we investigated the necessity of the relative position bias term [36,37] (see Section 3.1) in respect of the training results. This term encodes the relative position of the tokens among each other. As the training results show, the relative position bias does not have a large impact on the accuracy of our network. This is in contrast to Swin Transformer, where the accuracy strongly depends on the position bias. The consistent performance of our network without this term can be attributed to the convolutional operations in patch-embedding, which already perform implicit spatial encoding of the individual feature points. Even though the bias hardly contributes to the classification accuracy, it has an effect on the mAP of the object detection, which drops by −0.6.

**Feature-splitting.** We explored the impact of splitting features along channels before our novel parallel local-global self-attention Section 3.1. This is done primarily to save weights and complexity. As can be seen in Table 5, the network has 34% more parameters and 56% more FLOPs if the full number of channels is used for both local and global self-attention. This additional offset in terms of network complexity cannot be justified by the achieved accuracy values.

**Global attention window.** In Table 6, we present the impacts of the global window size and the downsampling method during patch sampling for global self-attention. First, we set the height $H_{gw}$ and the width $W_{gw}$ of the global-attention window to $(H_{gw},W_{gw})\in M\times M$ with M={7,10,14,18} and we report the ImageNet-1K Top-1 and Top-5 accuracy. The results reported in Table 6 show that with increase in the global window size, the model complexity in terms of FLOPS also increases. Furthermore, we observed a peak in accuracy when applying a window size of 14. Considering the moderate complexity in combination with the best Top-1 accuracy of 83.4, 14 was chosen as the general window size. In addition to evaluating the global window size, we also trained a model without global self-attention. Instead, we replaced self-attention with a pyramid-pooling module along the lines of PSPNet [60] and achieved a slightly lower accuracy (−0.3 Top-1) by using simple pooling operations.

**Patch-sampling.** In addition to examining the global window size, we also investigated the use of different pooling operations during patch sampling. For this purpose, we considered simple max-pooling, average-pooling, and the sum of both. The results are also listed in Table 6 and show that the combination of both achieves the best ImageNet Top-1 accuracy.

### 4.5. Computation Overhead Analysis

**Figure 5:**
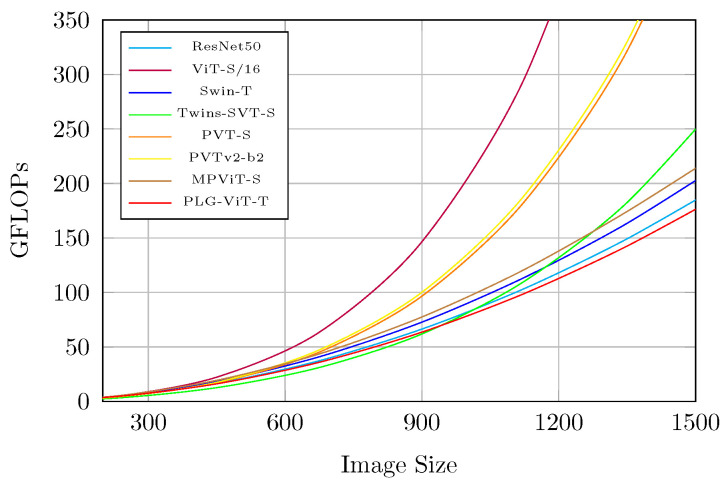
Complexity evaluation (GFLOPs) under different input sizes. The growth rate of GFLOPs: ViT-S/16 [14] > PVTv2-b2 [19] > PVT-S [13] > Twins-SVT-S [16] > MPViT-S [20] > Swin-T [12] > ResNet50 [1] > PLG-ViT-T (ours). All values are based on reproduced results.

Figure 5 illustrates the growth rate of the overall model complexity (GFLOPs) with increasing input size for several different models, including PLG-ViT *Tiny*, PVTv2-b2 [19], PVT-S [13], MPViT-S [20], Twins-SVT-S [16], ViT-S/16 [14], ResNet50 [1], and Swin-T [12]. The figure shows that as the input size increases, the growth rate of GFLOPs for PLG-ViT *Tiny* is much lower compared to PVT, Twins, and ViT, and is similar to that of ResNet50 and Swin. In particular, the standard self-attention of ViT and PVT shows a quadratic increase in complexity with respect to the resolution. The overall complexity of our network is much better compared to the other methods, especially for large image sizes, as shown in Figure 5. The runtime of our PLG-ViT depends on the network size and the hardware used. We achieved a maximum throughput of 352/213/160 frames per second for PLG-ViT *Tiny*, *Small*, and *Base* on a single NVIDIA RTX3060 GPU, respectively. These results suggest that our PLG-ViT is able to address the high computational overhead problem that arises due to the quadratic complexity increase of attention layers in “classical” ViT models.

### 4.6. Interpretability

**Figure 6:**
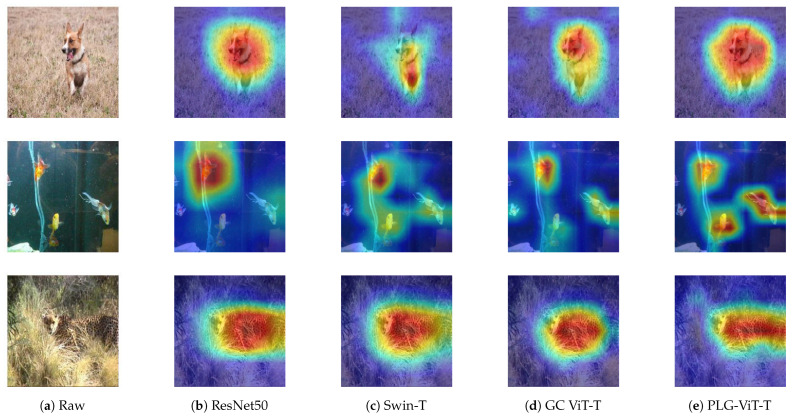
Visualization of the final activation with Grad-CAM [61]. (**a**): Input image; (**b**–**d**): SoTA [1,12,17]; (**e**) our network.

We used GradCAM [61] for the visualization of the final features. It generates heatmaps of an input image that highlight the regions of the image that are most important for the prediction. Figure 6 shows ImageNet-1K [21] val images and the final activation of ResNet50 [1], Swin-T [12], GC ViT-T [17], and our PLG-ViT-T. The GradCAM maps demonstrate the accurate object localization of our proposed method with the most intricate details. In particular, if multiple objects of the same class are present in the image, they are considered equally in the final prediction.

## 5. Conclusions

In this paper, we presented our Parallel Local-Global Vision Transformer (PLG-ViT) as a general-purpose backbone for image classification and dense downstream tasks. The core of our network is the eponymous parallel local-global self-attention, which separately extracts both local and global features and then fuses them to generate semantic representative features. We developed an effective feed-forward network, our CCF-FFN, which can further increase the effectiveness of our transformer encoder. In addition, due to the splitting of channels for the parallel local and global multihead self-attention, significant savings can be made in terms of the number of parameters and model complexity (e.g., FLOPs). Our Vision Transformer achieves state-of-the-art performance on COCO [22] object detection and ADE20K [41] semantic segmentation and outperforms most comparable networks. Furthermore, we also demonstrated the effectiveness of our network for use in diverse computer vision domains, such as autonomous driving [46], medical technology [45], and everyday situations [22,41].

## Figures and Tables

**Table 1:** Comparison of Top-1 image classification accuracy on the ImageNet-1K [21] validation set. Params refers to the number of parameters in millions and GFLOPs are calculated at a resolution of $224^{2}$. Our networks are highlighted in gray. 7 refers to a global window of size $7^{2}$. All values are taken from the official publications.

Method		Params (M)	GFLOPs	Top-1 (%)
	-50	25	4.1	76.1
ResNet [1]	-101	44	7.9	77.4
	-152	60	11.6	78.3
	-50-32x4d	25	4.3	77.6
ResNeXt [49]	-101-32x4d	44	8.0	78.8
	-101-64x4d	84	15.6	79.6
ViT [7]	-Base/16	86	17.6	77.9
DeIT [14]	-Small/16	22	4.6	79.9
	-Base/16	86	17.6	81.8
CrossViT [28]	-Small	26	5.6	81.0
	-Base	104	21.2	82.2
T2T-ViT [24]	-14	22	4.8	81.5
	-19	39	8.9	81.9
	-24	64	14.1	82.3
PVT [13]	-Small	24	3.8	79.8
	-Medium	44	6.7	81.2
	-Large	61	9.8	81.7
PVTv2 [19]	-B2	25	4.0	82.0
	-B3	45	6.9	83.2
	-B4	62	10.1	83.6
DPT [50]	-Small	26	4.0	81.0
	-Medium	46	6.9	81.9
Twins-PCPVT [16]	-Small	24	3.8	81.2
	-Base	44	6.7	82.7
	-Large	61	9.8	83.1
Twins-SVT [16]	-Small	24	2.9	81.7
	-Base	56	8.6	83.2
	-Large	99	15.1	83.7
CoAtNet [47]	-0	25	4.2	81.6
	-1	42	8.4	83.3
	-2	75	15.7	84.1
Swin [12]	-Tiny	29	4.5	81.3
	-Small	50	8.7	83.2
	-Base	88	15.5	83.5
DAT [18]	-Tiny	29	4.6	82.0
	-Small	50	9.0	83.7
	-Base	88	15.8	84.0
PoolFormer [51]	-S24	21	3.6	80.3
ConvNeXt [9]	-Tiny	29	4.5	82.1
	-Small	50	8.7	83.1
	-Base	89	15.4	83.8
Focal [15]	-Tiny	29	4.9	82.2
	-Small	51	9.1	83.5
	-Base	90	16.0	83.8
CSwin [52]	-Tiny	23	4.3	82.7
	-Small	35	6.9	83.6
	-Base	78	15.0	84.2
MPViT [20]	-XSmall	11	2.9	80.9
	-Small	23	4.7	83.0
	-Base	75	16.4	84.3
HorNet [53]	-Tiny	22	4.0	82.8
	-Small	50	8.8	83.8
	-Base	87	15.6	84.2
VAN [54]	-B2	27	5.0	82.8
	-B3	45	9.0	83.9
	-B4	60	12.2	84.2
GC ViT [17]	-Tiny	28	4.7	83.4
	-Small	51	8.5	83.9
	-Base	90	14.8	84.4
PLG-ViT (ours)	-Tiny-7	27	4.0	82.9
	-Tiny	27	4.3	83.4
	-Small	52	8.6	84.0
	-Base	91	15.2	84.5

**Table 2:** COCO [22] instance segmentation results with Mask RCNN [56]. All models were trained with a 3× schedule and multi-scale inputs. GFLOPs were calculated with image size (1280, 800). Our networks are highlighted in gray. All values are taken from the official publications.

Backbone	Param (M)	GFLOPs	${\mathrm{AP}}^{\mathrm{box}}$	${\mathrm{AP}}_{50}^{\mathrm{box}}$	${\mathrm{AP}}_{75}^{\mathrm{box}}$	${\mathrm{AP}}^{\mathrm{mask}}$	${\mathrm{AP}}_{50}^{\mathrm{mask}}$	${\mathrm{AP}}_{75}^{\mathrm{mask}}$
ResNet50 [1]	44.2	260	41.0	61.7	44.9	37.1	58.4	40.1
PVT-S [13]	44.1	245	43.0	65.3	46.9	39.9	62.5	42.8
Swin-T [12]	47.8	264	46.0	68.1	50.3	41.6	65.1	44.9
ConvNeXt-T [9]	48.0	262	46.2	67.9	50.8	41.7	65.0	44.9
DAT-T [18]	48.0	272	47.1	69.2	51.6	42.4	66.1	45.5
Focal-T [15]	48.8	291	47.2	69.4	51.9	42.7	66.5	45.9
MPViT-S [20]	43.0	268	48.4	70.5	52.6	43.9	67.6	47.7
GC ViT-T [17]	48.0	291	47.9	70.1	52.8	43.2	67.0	46.7
PLG-ViT-T	46.3	250	48.2	69.5	53.0	42.9	66.9	46.1
ResNet101 [1]	63.2	336	42.8	63.2	47.1	38.5	60.1	41.3
ResNeXt101-32x4d [49]	62.8	340	44.0	64.4	48.0	39.2	61.4	41.9
PVT-M [13]	63.9	302	44.2	66.0	48.2	40.5	63.1	43.5
Swin-S [12]	69.1	354	48.5	70.2	53.5	43.3	67.3	46.6
DAT-S [18]	69.1	378	49.0	70.9	53.8	44.0	68.0	47.5
Focal-S [15]	71.2	401	48.8	70.5	53.6	43.8	67.7	47.2
PLG-ViT-S	71.2	335	49.0	70.2	53.8	43.5	67.2	46.5
ResNeXt101-64x4d [49]	102.0	493	44.4	64.9	48.8	39.7	61.9	42.6
PVT-L [13]	81.0	364	44.5	66.0	48.3	40.7	63.4	43.7
Swin-B [12]	107.0	496	48.5	69.8	53.2	43.4	66.8	46.9
Focal-B [15]	110.0	533	49.0	70.1	53.6	43.7	67.6	47.0
MPViT-B [20]	95.0	503	49.5	70.9	54.0	44.5	68.3	48.3
PLG-ViT-B	110.5	461	49.5	70.6	54.0	43.8	67.7	47.3

**Table 3:** Object detection results for 3 vision benchmarks [22,45,46] from diverse domains. Comparison of our PLG-ViT-T with Swin-T [12], PVTv2-b2 [19], ConvNeXt-t [9], and ResNet50 [1] using Faster RCNN [55] and RetinaNet [33]. FLOPs were calculated with image size (1280, 800). All models were trained with a single-scale input and 1× scheduler and the best results are highlighted in bold. All values are based on reproduced results.

		Faster RCNN [55]					RetinaNet [33]				
		Resnet50 [1]	ConvNeXt-T [9]	Swin-T [12]	PVTv2-b2 [19]	PLG-ViT-T	Resnet50 [1]	ConvNeXt-T [9]	Swin-T [12]	PVTv2-b2 [19]	PLG-ViT-T
Param (M)		41.5	45.5	45.2	42.4	43.6	37.4	38.8	38.5	35.1	36.7
GFLOPs		207	209	211	184	197	239	243	245	218	231
**COCO** [22]	AP	37.4	43.5	42.0	44.9	**45.1**	36.5	43.4	41.9	44.6	**44.8**
	AP^50^	58.1	65.8	64.8	**67.2**	**67.2**	55.4	64.1	62.8	65.6	**65.7**
	AP^75^	40.4	47.7	45.9	49.0	**49.2**	39.1	46.8	44.7	47.6	**48.3**
	AP^S^	21.2	26.8	26.1	**29.2**	27.8	20.4	26.9	25.4	27.4	**28.0**
	AP^M^	41.0	47.2	45.5	48.6	**48.7**	40.3	47.8	45.5	48.8	**49.0**
	AP^L^	48.1	56.6	55.5	58.8	**59.4**	48.1	56.6	55.1	**58.6**	**58.6**
**BDD100K** [46]	AP	31.0	33.3	32.1	32.9	**33.7**	28.6	**33.0**	31.8	32.4	**33.0**
	AP^50^	55.9	59.5	58.8	59.0	**60.3**	52.1	**59.0**	57.4	58.4	58.9
	AP^75^	29.4	31.5	29.8	31.4	**32.6**	26.6	31.1	29.8	30.8	**31.4**
	AP^S^	14.7	16.1	15.2	16.1	**16.4**	10.6	13.9	13.1	13.9	**14.2**
	AP^M^	36.0	37.5	37.0	37.2	**38.7**	34.6	38.5	37.3	37.9	**38.6**
	AP^L^	50.9	55.2	53.7	54.3	**54.6**	49.6	**55.9**	55.1	55.1	55.5
**AGAR** [45]	AP	55.4	54.3	56.7	54.0	**58.4**	49.3	52.9	55.6	55.3	**56.5**
	AP^50^	79.1	79.3	79.3	79.9	**80.3**	76.6	80.6	80.7	**81.4**	80.8
	AP^75^	65.2	61.5	67.5	61.3	**69.6**	55.9	58.5	65.1	62.6	**66.7**
	AP^S^	2.4	3.4	2.8	3.7	**4.6**	8.2	11.5	12.2	13.8	**13.9**
	AP^M^	40.2	39.5	42.1	39.6	**44.0**	29.6	33.3	37.7	36.9	**38.2**
	AP^L^	62.3	60.7	63.1	60.5	**64.4**	57.8	60.6	62.7	61.6	**63.9**

**Table 4:** Validation results for semantic segmentation with UPerNet [58] on the ADE20K val-set [41]. GFLOPS were calculated using images of size (2048, 512). The last two columns show single- and multi-scale (ms) evaluations of the mIoU. Our networks are highlighted in gray. All values are taken from the official publications.

Backbone	Params (M)	GFLOPs	mIoU	mIoU^ms^
Swin-T [12]	60	945	44.5	45.8
DAT-T [18]	60	957	45.5	46.4
ConvNeXt-T [9]	60	939	46.1	46.7
Twins-SVT-S [16]	54	896	46.2	47.1
Focal-T [15]	62	998	45.8	47.0
MPViT-S [20]	52	943	48.3	N/A
GC ViT-T [17]	58	947	47.0	N/A
PLG-ViT-T	56	925	46.4	47.2
Swin-S [12]	81	1038	47.6	49.5
DAT-S [18]	81	1079	48.3	49.8
ConvNeXt-S [9]	82	1027	48.6	49.6
Twins-SVT-B [16]	88	1005	47.7	48.9
Focal-S [15]	85	1130	48.0	50.0
GC ViT-S [17]	84	1163	48.3	N/A
PLG-ViT-S	83	1014	48.0	48.6
Swin-B [12]	121	1188	48.1	49.7
DAT-B [18]	121	1212	49.4	50.6
ConvNeXt-B [9]	122	1170	48.7	49.9
Twins-SVT-L [16]	133	1134	48.8	50.2
Focal-B [15]	126	1354	49.0	50.0
MPViT-S [20]	105	1186	50.3	N/A
GC ViT-B [17]	125	1348	49.0	N/A
PLG-ViT-B	125	1147	49.9	50.7

**Table 5:** Ablation study on the effectiveness of the components in PLG-ViT on the tasks of classification, detection, and instance segmentation. Mask RCNN [56] with a 1× scheduler and ms-training was used for evaluation on COCO [22]. w/o PLG-SA: only local window self-attention; w/o CCF-FFN: replace CCF-FFN with linear MLP; w/o Conv-PE: remove overlapping patch-embedding and convolutional downsampling; Swin Config: use standard config of Swin [12]; w/o rel. pos.: remove relative position bias term; w/o ch. split: PLG-SA without previous splitting along the channels.

Modules	Param (M)	GFLOPs	ImageNet-1K		COCO	
			Top-1	Top-5	AP^box^	AP^mask^
w/o PLG-SA	28.8	4.9	83.2	96.5	46.0	41.2
w/o CCF-FFN	26.3	4.2	82.6	96.2	43.7	40.1
w/o Conv-PE	25.6	4.2	83.3	96.5	45.7	40.8
Swin Config	30.0	4.7	82.9	96.3	44.6	40.7
w/o rel. pos.	26.5	4.3	83.3	96.4	45.6	40.8
w/o ch. split	35.7	6.7	83.7	96.7	46.3	41.5
PLG-ViT	26.6	4.3	83.4	96.4	46.2	41.3

**Table 6:** Ablation study of global self-attention on ImageNet-1K [21]. In the first part, the influence of the global window size was determined. PPM refers to the pyramid-pooling module [60], which replaced our global self-attention in this case. In the second part, the impact of pooling on patch-sampling was determined.

Global Window Size Experiments				
**Win-Size**	**Param (M)**	**GFLOPs**	**Top-1 (%)**	**Top-5 (%)**
7	26.6	4.0	82.9	96.3
10	26.6	4.1	83.1	96.3
14	26.6	4.3	83.4	96.4
18	26.7	4.8	83.2	96.4
PPM	26.3	4.1	83.1	96.4
**Patch Sampling Experiments**				
**Pooling**	**Params (M)**	**GFLOPs**	**Top-1 (%)**	**Top-5 (%)**
Max	26.6	4.3	83.2	96.4
Avg	26.6	4.3	83.1	96.3
Max + Avg	26.6	4.3	83.4	96.4

**Figure A1:**
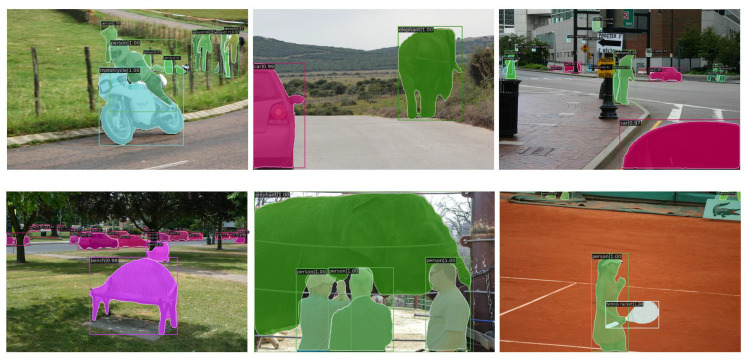
Examples of our PLG-ViT with Mask RCNN [56] on COCO [22].

**Figure A2:**
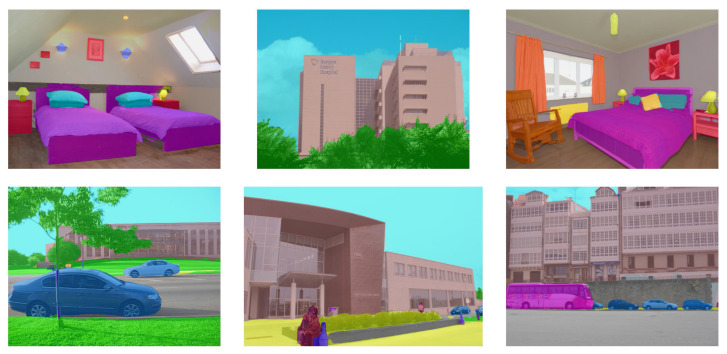
Examples of our PLG-ViT with UPerNet [58] on ADE20K [41].

**Figure A3:**
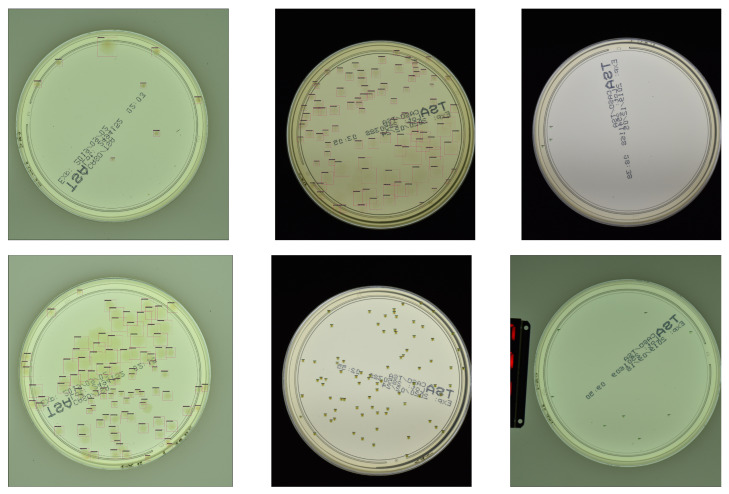
Examples of our PLG-ViT with Faster RCNN [55] on AGAR [45].

**Figure A4:**
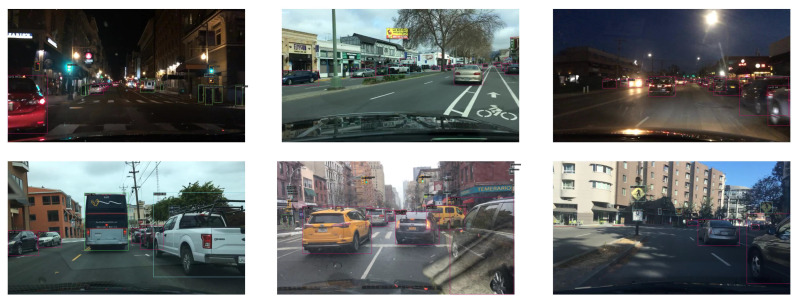
Examples of our PLG-ViT with Faster RCNN [55] on BDD100K [46].

## Data Availability

Restrictions apply to the availability of these data. ImageNet data were obtained from [21] and are available at https://www.image-net.org/index.php (accessed on 8 February 2023). COCO data were obtained from [22] and are available at https://cocodataset.org/ (accessed on 8 February 2023). ADE20k data were obtained from [41] and are available at https://groups.csail.mit.edu/vision/datasets/ADE20K/ (accessed on 8 February 2023). AGAR data were obtained from [45] and are available at https://agar.neurosys.com/ (accessed on 8 February 2023). BDD100k data were obtained from [46] and are available at https://bdd-data.berkeley.edu/ (accessed on 8 February 2023).

## Appendix A. Detailed Architectures

We explained the configuration of our PLG-ViT in Section 3.3. This chapter only serves to describe it in more detail with the help of Table A1. Similar to comparable methods [12,13,33], we consider three network configurations. These three proposed models are PLG-ViT *Tiny*, *Small*, and *Base*, as shown in Table A1. Furthermore, the *Tiny* and *Small* versions are only 0.25× and 0.5× the size and computational complexity of PLG-ViT *Base*. In the table, we list all four transformer stages and the CNN stem as *stage 0*, at the beginning. With the CNN stem, we reduced the spatial dimension of the input image by a stride of s=4 and increased the channel size to c1 to {64,96,128} for *Tiny*, *Small*, and *Base*, respectively. For stages 1,2, and 3, we specify in the table the parameters for local-window (*lwsa*) and global-window self-attention (*gwsa*). The first number in each curly bracket indicates the window size. As can be seen, we set the local and global window sizes to 7 and 14, respectively. A more detailed analysis of the impact of the global window size can be found in the ablation study (see Section 4.4). The second number in the brackets refers to the number of heads. We set the dimension *d* of each head to 32 for *Base* and *Tiny*, and to 24 channels for PLG-ViT *Small*. The expansion ratio of CCF-FFN was α=4 for all experiments. Furthermore, the number of repetitions of each transformer layer can be seen behind each square bracket. Downsampling takes place after each transformer stage. The spatial resolution is reduced by 2 and the number of features is increased by 2. The fourth and final transformer stage is different from the first three. Here, due to the low resolution of the features, only a local window is generated, which in the case of an input resolution of $224^{2}$ is equivalent to a global window. The choice of hyperparameters is supported by the evaluation presented in Section 4.4.

**Table A1:** Model configurations for our PLG-ViT. There are three configurations introduced: PLG-ViT *Tiny*, PLG-ViT *Small*, and PLG-ViT *Base*, with different model capacities.

	Output Size	Layer Name	PLG-ViT *Tiny*			PLG-ViT *Small*			PLG-ViT *Base*		
Stage 0	56×56	CNN-Stem	stride=4;c1=64			stride=4;c1=96			stride=4;c1=128		
Stage 1	56×56	PLG-Block	[	lwsa={7,1}	] ×3	[	lwsa={7,2}	] ×3	[	lwsa={7,2}	] ×3
				gwsa={14,1}			gwsa={14,2}			gwsa={14,2}	
Stage 2	28×28	CNN-Down	stride=2;c2=128			stride=2;c2=192			stride=2;c2=256		
		PLG-Block	[	lwsa={7,2}	] ×4	[	lwsa={7,4}	] ×3	[	lwsa={7,4}	]×3
				gwsa={14,2}			gwsa={14,4}			gwsa={14,4}	
Stage 3	14×14	CNN-Down	stride=2;c3=256			stride=2;c3=384			stride=2;c3=512		
		PLG-Block	[	lwsa={7,4}	] ×16	[	lwsa={7,8}	] ×16	[	lwsa={7,8}	] ×16
				gwsa={14,4}			gwsa={14,8}			gwsa={14,8}	
Stage 4	7×7	CNN-Down	stride=2;c4=512			stride=2;c4=768			stride=2;c4=1024		
		PLG-Block	[	lwsa={7,16}	] ×4	[	lwsa={7,24}	] ×3	[	lwsa={7,32}	] ×3

## Appendix B. Detailed Experimental Settings

We performed comprehensive experiments on the benchmarks ImageNet-1K [21] for image classification, COCO [22] for object detection and instance segmentation, and ADE20K [41] for semantic segmentation. Furthermore, we also investigated the effectiveness of our network in different application domains using the two datasets BDD100k [46] and AGAR [45]. In the following, we explain the different training strategies in more detail.

### Appendix B.1. Image Classification on ImageNet-1K

For the task of image classification (see Section 4.1) we used ImageNet-1K [21], which consists of 1.28M images for training and 50K images for validation from 1000 classes. The classification task is solved by a combination of global average pooling of the output features of the last transformer stage and a subsequent linear classifier. For evaluation of the Top-1 accuracy, we report the results for a single crop.

Our training mostly follows DeIT [14] and Swin Transformer [12]. For all models, the input resolution of $224^{2}$ is used. During the training, we use the AdamW optimizer [62] with a momentum of 0.9 and a total batch-size of 1024 with a basic learning rate of $1\times 10^{-3}$. For a fair comparison with most competing methods [12,14,15,16,17,18,52], we also train for only 300 epochs. Gradient clipping with a max norm of 1 and a weight decay of 0.05 are used. Furthermore, we employ a cosine decay learning rate scheduler with 20 epochs of linear warm-up to decrease the learning rate to $1\times 10^{-7}$ during the 300 epochs of training. To avoid overfitting, we mostly follow DeIT’s [14] augmentation strategy. This includes RandAugment [63], Cutmix [64], Mixup [65], random erasing [66], and stochastic depth [67]. The degree of stochastic depth augmentation is increased for larger models, i.e., 0.2,0.3,0.5 for *Tiny*, *Small*, and *Base*, respectively. Due to the fact that the ineffectiveness has already been demonstrated in other work [12], we omit repeated augmentation [68] and exponential moving average (EMA) [69]. All training was performed on eight NVIDIA A100 GPUs and required between 38 and 72 h, depending on the network size (similar to Swin Transformer). The results can be found in Table 1.

### Appendix B.2. Object Detection and Instance Segmentation

For training and evaluation of object detection and instance segmentation as described in Section 4.2, we employ mainly the COCO [22] dataset, which contains 118K training and 5K validation images of everyday objects from 80 classes. Our pre-trained models are used as the backbone for the typical frameworks of Faster RCNN [55] and RetinaNet [33] for pure object detection, and Mask RCNN [56] for instance segmentation.

Like most competing methods [12,16,17,33], we also follow the two standard schedules, a 1× schedule with 12 and a 3× schedule with 36 training epochs. For the 1× schedule we resize the shorter side of the image to 800 and keep the longer side to a maximum of 1333. For the 3× schedule we perform multi-scale training and vary the shorter side between 480 and 800, and keep the longer side almost to 1333. We use AdamW [23] as the optimizer with a total batch size of 16 and an initial learning rate of $1\times 10^{-4}$ with a weight decay of 0.05. The implementation of all methods is based on the MMDetection Framework [57]. All training was performed on four NVIDIA A100 GPUs. The results can be found in Table 2.

To investigate the network performance for object detection in different domains with different characteristics (see Section 4.2), we trained our PLG-ViT-T as the backbone of RetinaNet and Faster RCNN on the three dataset COCO, BDD100K [46], and AGAR [45]. The BDD100K dataset shows multiple scenarios from autonomous driving and contains 70K images for training and 10K images for validation from 10 classes. Furthermore, the AGAR dataset from the field of medical technology was used for additional evaluation. This dataset contains high-resolution images of five different kinds of bacteria that were grown on a culture medium of agar plates. AGAR contains approximately 5K training and 2K validation images. In the context of this experiment, we performed single-scale training of 12 epochs with the described settings of the 1× scheduler. Only the resolution of the images differed. For BDD10K, we set the size of the images between 1280 and 720 and for AGAR we limited the longest side to 1536 pixels. All training was performed on two NVIDIA A100 GPUs. The results can be found in Table 3.

### Appendix B.3. Semantic Segmentation on ADE20K

For benchmarking our results of semantic segmentation (see Section 4.3), we used the ADE20K [41] dataset, which contains 20K images for training and 2K images for validation from 150 classes. We employed our pre-trained model as the backbone and utilized UPerNet [58] in MMSegmentation [59] as our framework. We trained the networks for 160K iterations with an input size of 512×512 and a total batch size of 16. The AdamW optimizer was used with an initial learning rate of $6\times 10^{-5}$, a weight decay of 0.01, and a polynomial decay scheduler with a linear warmup of 1500 iterations. Further, the default settings of MMSegmentation were used for augmentation. These are random horizontal flipping, random rescaling within ratio range [0.5, 2.0], and random photometric distortion. In addition, we used stochastic depth with a ratio of 0.2 for all our models. All training was performed on two NVIDIA A100 GPUs. The results can be found in Table 4.

## Appendix C. Visual Examples

In Figure A1, Figure A3, and Figure A4, we show some qualitative results of our PLG-ViT as the backbone for object detection (COCO [22], BDD100K [46], and AGAR [45]) and instance segmentation (COCO). Furthermore, we present some qualitative results (see Figure A2) of semantic segmentation (ADE20K [41]). These results demonstrate our network’s ability to extract powerful features for different downstream vision tasks.
